# Mitochondria: a crucial factor in the progression and drug resistance of colorectal cancer

**DOI:** 10.3389/fimmu.2024.1512469

**Published:** 2024-12-23

**Authors:** Ying Zhao, Xiaomin Guo, Li Zhang, Dongwei Wang, Yan Li

**Affiliations:** ^1^ Department of Pharmacy, Jinan Fifth People’s Hospital, Jinan, China; ^2^ Department of Surgery, Jinan Second People’s Hospital, Jinan, China; ^3^ Pharmacy Department, Community Health Service Centre of Guyunhu Street, Jinan, China; ^4^ Department of Proctology, Jinan Huaiyin People’s Hospital, Jinan, China; ^5^ Department of Pharmacy, Jinan Fourth People’s Hospital, Jinan, China

**Keywords:** colorectal cancer, multimodal analysis techniques, mitochondria, chemotherapy resistance, treatment strategies

## Abstract

Colorectal cancer (CRC), as one of the malignant tumors with the highest incidence and mortality rates worldwide in recent years, originating primarily from the mucosal tissues of the colon or rectum, and has the potential to rapidly develop into invasive cancer. Its pathogenesis is complex, involving a multitude of factors including genetic background, lifestyle, and dietary habits. Early detection and treatment are key to improving survival rates for patients with CRC. However, the pervasive problem is that patients can become severely resistant to treatment, which greatly increases the complexity and challenge of treatment. Therefore, unraveling and overcoming the resistance of CRC has become a focus of research. Mitochondria, the energy centers of the cell, play a crucial role in cellular metabolism, energy supply, and the apoptosis process. In CRC, Mitochondrial dysfunction not only impairs normal cell function but also promotes tumor resistance. Therefore, a deep understanding of the relationship between mitochondrial dysfunction and the mechanisms of CRC development, as well as the mechanisms by which it promotes resistance to chemotherapy drugs, is crucial for the development of targeted therapies, enhancing drug efficacy, and improving treatment outcomes and quality of life for patients.

## 1 Introduction

Mitochondria are renowned not merely as the cellular powerhouses for ATP production but also play pivotal roles in synthesis, metabolism, signal transduction, and maintaining cellular homeostasis under both physiological and pathological conditions (1–3). They are crucial for cell growth, development, and the regulation of cell death. Since Otto Warburg’s 1930s proposition linking mitochondrial function to cancer progression, the role of mitochondria has been extensively studied. Central to ATP generation and biosynthesis of macromolecules, mitochondria’s involvement in cancer through bioenergetics, dynamics, and signaling is increasingly acknowledged (4–6). It has been found that metastatic tumor cells and cancer stem cells, drive tumorigenesis through the oxidative phosphorylation (OXPHOS) metabolic pathway. Mitochondrial metabolism is critical for tumorigenesis and development. Interestingly, recent studies have found that: tumor cells develop severe resistance to chemotherapy via OXPHOS (7, 8). The effect of mitochondria on tumorigenesis and progression varies depending on the type of cancer, differences in the tumor microenvironment and genetic, environmental and other factors (9). Mitochondria play a critical role during cancer chemotherapy as sensors of cellular sensory stress. The main mechanisms that have been identified include disturbances in mitochondrial dynamics, mitochondrial DNA mutations, dysregulation of Ca ion dynamics, and abnormalities in the autophagy pathway, all of which are crucial for improving the efficacy of tumor chemotherapy and finding specific mechanisms of resistance to cancer chemotherapy (10–12).

CRC is one of the most prevalent cancers in the world. It ranks third in the United States and fourth in China, causing a huge global health and economic burden (13). In recent years, despite significant advances in chemotherapy, molecularly targeted therapy, and immunotherapy, the mortality rate of CRC remains high. The main reasons for this include the susceptibility of CRC to metastasis and recurrence, as well as resistance to treatment (14). Notably, approximately 20% of CRC patients have metastasis at the time of diagnosis, and the five-year survival rate of these patients is less than 20% (15–17). Therefore, it is particularly important to explore new therapeutic targets for drug resistance and to deepen the understanding of cancer mechanisms (18, 19). Mitochondria play a central role in the regulation of cellular metabolism, energy production, signaling, and cell death, and are essential for maintaining the normal and transformed state of cells (20, 21). Numerous studies have shown that mitochondrial dysfunction contributes to chemoresistance in CRC at different levels, including altering the metabolic pattern of cancer cells, activating anti-apoptotic mechanisms, and affecting cellular responses to chemotherapeutic agents (8, 22). In-depth study of the mitochondria and its histology in the occurrence and development of CRC, as well as the mitochondria as a specific target to explore how to overcome the resistance to chemotherapy, will provide a key direction for the development of novel therapeutic strategies in the future.

## 2 Mitochondrial dysfunction drives CRC onset, progression

### 2.1 Mitochondrial dynamics drive CRC genesis

Mitochondria are central to the process of CRC genesis and development. Mitochondrial dynamics are mainly divided into mitochondrial fission and fusion. When mitochondrial dynamics are disturbed, it directly promotes the proliferation, invasion and migration of CRC cells. One of the most important factors affecting mitochondrial dynamics is the inhibition of the expression of the mitochondrial fission protein dynamin-related protein 1 (Drp1). Abnormal expression of Drp1 triggers a series of changes, including mitochondrial elongation, decreased mitochondrial membrane potential (MMP), and increased cytochrome C release. All of these factors directly drive CRC proliferation and metastasis (23, 24). Importantly, Drp1 orchestrates fatty acid (FAs) metabolism and the morphological integrity of mitochondria within CRC cells (25). Fatty acids treatment not only triggers mitochondrial bifurcation through the phosphorylation of Drp1 at Ser616 and its intensified interaction with Mff but also catalyzes FAs oxidation and stimulates the Wnt/β-catenin signaling cascade, thereby propelling CRC cell proliferation and invasive behavior. Furthermore, the dissection of mitochondrial dynamics extends to glucose metabolism reengineering, enhancing CRC cell proliferation, invasion, and migratory capacity (26). Specifically, in CRC cells bearing the BRAFV600E mutation, the activation of the MEK/ERK signaling axis facilitates Drp1 phosphorylation at Ser616, culminating in mitochondrial fragmentation and subsequent tumor progression (27). The aberrant expression of Mitochondrial Fission Regulator 2 (MTFR2) in CRC tissue underscores its regulatory role in mitochondrial segmentation (23, 28). Targeted knockout of MTFR2 in HCT116 cells can significantly reduce the proliferation, invasion, and migration of CRC cells. Further research has found that the above processes are primarily mediated by the dysregulation of the miR-27a/FOXJ3 signaling pathway, leading to abnormalities in mitochondrial biogenesis. This results in a reduction in mitochondrial size and number, promoting the emergence of a phenotype characterized by punctate mitochondria (29). Furthermore, overexpression of deubiquitinating enzyme 6A (OTUD6A) promotes deubiquitination of Drp1, which increases proliferation and cloning of CRC cells. In contrast, knockdown of OTUD6A extends the length of mitochondria and inhibits their fragmentation, which significantly inhibits the proliferation of CRC cells (30). The above studies elucidated the critical role of the regulation of mitochondrial dynamics in CRC pathogenesis. Abnormal mitochondrial fission and fusion lead to mitochondrial energy metabolism disorders and signal transduction abnormalities, which in turn promote CRC (31).

### 2.2 The role of mitochondrial biogenesis in CRC progression

Mitochondrial biogenesis influences CRC progression through a variety of molecular mechanisms and signaling pathways, and plays complex and multidirectional biological roles in CRC progression. Proteins such as Peroxisome proliferator-activated receptor-gamma coactivator (PGC)-1α, TFAM, and SIRT play key roles in regulating mitochondrial number and function, which in turn, directly regulate the energy metabolism, proliferation, and drug resistance of CRC cells (32). PGC-1α can promote mitochondrial biogenesis and plays a double-edged role in regulating tumor progression by enhancing the oxidative phosphorylation capacity of cells (33). On the one hand, PGC-1α activation leads to an increase in the number of mitochondria, which inhibits CRC progression by decreasing antioxidant enzyme activity increasing oxidative stress and promoting apoptosis. In CRC cells, PGC-1α induced mitochondrial death through the BAX signaling pathway, thereby promoting apoptosis and inhibiting tumor growth. Interestingly, another study confirmed that PGC-1α enhances the expression of genes involved in oxidative phosphorylation and TCA cycle, increases fatty acid synthesis, and promotes energy supply, thus supporting the rapid proliferation of CRC cells (34). Furthermore, TFAM is crucial for regulating the replication and stability of mitochondrial DNA. Research has found that overexpression of TFAM can promote CRC proliferation by upregulating the classic NF-κB pathway and inducing the production of ROS. Conversely, mutations in TFAM or a decline in its activity can reduce mtDNA content, affect mitochondrial function, and thus promote tumor development (35, 36). The multiple roles of SIRT proteins: SIRT proteins, through their NAD+-dependent deacetylase activity, participate in the fine-tuning of mitochondrial metabolism and function, exerting dual regulatory effects on CRC development. For example, SIRT3 can promote tumor growth by enhancing the deacetylation and thus activity of SHMT2, but it can also exert anti-tumor effects by increasing SOD2 activity and reducing ROS production (37, 38). SIRT4 inhibits tumor growth by suppressing glutamate metabolism, whereas the role of SIRT5 is more complex, involving the regulation of various metabolic pathways and enzyme activities, potentially affecting CRC cell proliferation and survival by impacting key steps in nucleotide synthesis and the TCA cycle (39). In summary, mitochondrial biogenesis and its regulatory mechanisms play complex and varied roles in CRC, involving multiple aspects of cell energy metabolism, apoptosis, DNA stability, and oxidative stress. A deeper understanding of these mechanisms offers the potential for discovering new therapeutic targets, paving the way for the development of novel treatment strategies for CRC.

## 3 Crosstalk between microbes and mitochondria

In recent years, scientists have found that the microbiome plays a crucial role in promoting the occurrence and development of CRC (40, 41). At the same time, the close relationship between the microbiome and mitochondria of tumor cells has been gradually revealed. Interestingly, the mitochondrial changes in CRC are significantly different from those observed in other cancers, especially in terms of mtDNA cloning mutations (8, 42). Interestingly, the frequency of these mutations is significantly lower in CRC cells compared to adjacent non-tumorous tissues. Further investigation into this phenomenon revealed a metabolic shift in CRC cells from oxidative phosphorylation (OXPHOS) to anaerobic glycolysis. Additionally, research has confirmed that due to the strong mitochondrial genome stability, there is less mtDNA damage caused by ROS. Thus, studies on the microbiome have mainly focused on mitochondrial metabolic pathways related to CRC. Under hypoxic conditions, the microbiome activates the mitochondrial protease OMA1, which promotes mitochondrial ROS production, upregulates HIF-1α, and promotes glycolysis in CRC cells, thereby facilitating CRC proliferation (43). It has been discovered that the Fusobacterium nucleatum adhesin A (FadA) on the surface of F. nucleatum activates the PI3K/Akt/mTOR signaling pathway, promoting intestinal inflammation and CRC cell proliferation. Anaerobic streptococci, by upregulating the expression of TLR2 and TLR4 signals, induce the production of intracellular ROS and promote the biometabolism of cholesterol, thereby providing ample energy for the development of CRC (44). Bacterial virulence factors have been proven to induce mtDNA mutations and promote the development of CRC by regulating endogenous apoptosis pathways. For instance, virulence factors produced by Escherichia coli have been shown to promote the occurrence and progression of CRC. Khan and colleagues, using artificial intelligence methods, identified 87 proteins from E. coli. These proteins target the mitochondria of host intestinal mucosal cells and participate in the mechanisms of CRC development (45). Propionibacterium produces short-chain fatty acids, which by inhibiting the mitochondrial membrane potential and inducing ROS production in CRC cells, promote their proliferation (46). The above findings confirm that: interactions between the microbiome and mitochondria play a key role in the development of CRC.

By targeting the microbiota, it can be a key strategy for CRC treatment. The findings suggest that certain bacterial taxa are highly beneficial in regulating mitochondrial function. They play a key role in CRC therapy by altering mitochondrial dynamics and mitochondrial metabolism. In a mouse model of diabetes induced through a Western dietary regimen, specific Lactobacillus strains increased the expression of genes related to mitochondrial function (47). This upregulation of genes coincided with alterations in mitochondrial structure and morphology and ameliorated to a great extent the metabolic dysregulation caused by the Western dietary pattern (48). In addition, post-biotic compounds derived from Lactobacillus casei have been shown to possess antioxidant properties that ameliorate hepatic mitochondrial dysfunction (49). Microbiota’s metabolic byproducts, such as succinate, have been demonstrated to facilitate the expression of genes regulating the tricarboxylic acid (TCA) cycle, reducing inflammatory responses in murine models of enteritis. Concurrently, short-chain fatty acids (SCFAs), for instance, acetate and butyrate, play a crucial role in the modulation of mitochondrial metabolic pathways (50). In mouse models of obesity and insulin resistance, administration of sodium butyrate significantly attenuated ROS generation and enhanced mitochondrial functionality within the hepatic domain. Therapeutic interventions utilizing acetate and butyrate demonstrated prophylactic effects against mitochondrial dysfunction and conferred resistance to metabolic stress induced by streptozotocin, a facet of paramount importance for human islets and β-cells (51). Given that SCFAs represent primary fermentation end-products of dietary fibers by gut microbiota, augmenting dietary intake of both soluble and insoluble fibers could potentially enhance SCFA biosynthesis, thereby facilitating mitochondrial function regulation. SCFAs derived from the microbiota, such as butyrate, are implicated in the regulation of host gene expression associated with intestinal homeostasis and carcinogenesis through the modulation of colonic epithelial miRNA profiles, illustrating intricate microbe-host cellular interactions. Specific microbial taxa and their metabolites, impacting host mitochondrial pathways, hold promise for therapeutic intervention, exemplified by bacterial mutants that prolong lifespan via enhanced secretion of the polysaccharide colanic acid (CA) (52). This polysaccharide modulates host mitochondrial dynamics and the mitochondrial unfolded protein response (UPRmt). Alterations within mitochondrial parameters may reciprocally influence the composition of the microbiota, with a documented inverse relationship between mitochondrial ROS output and microbial species diversity. These insights propose that modifications in mitochondrial redox balance and associated ROS generation could represent a novel therapeutic strategy for conditions linked to dysbiosis (53). In prospective preclinical trials using mitochondria-targeted antioxidants, such as MitoTEMPO and MitoQ: findings have shown their potential to reduce inflammation, alleviate colitis severity and modulate the gut microbiome (54).

In conclusion, mitochondrial-microbial interactions show a complex network of mechanisms in regulating the progression of CRC. These mechanisms involve shifts in metabolic pathways, alterations in mitochondrial DNA stability. By targeting microbes as well as metabolites to regulate mitochondrial function, the proliferation and progression of CRC cells is inhibited. In the future, unraveling the intricate relationship between the microbiota and mitochondria will help us gain a deeper understanding of the pathogenesis of CRC and provide potential avenues for the development of targeted therapies and interventions.

## 4 Mitochondrial dysfunction promotes chemotherapy resistance in CRC

### 4.1 Relationship between mitophagy and chemotherapy resistance

Mitophagy is an evolutionarily conserved autophagic process that selectively eliminates damaged mitochondria to maintain cellular homeostasis. This process is regulated via both ubiquitin-dependent and non-ubiquitin-dependent pathways, with the latter being mediated by autophagy receptors (48). In the ubiquitin-dependent pathway, PINK1 and Parkin are critical proteins. The PINK1-Parkin axis promotes the ubiquitination of mitochondrial outer membrane proteins (e.g., Mfn1, Mfn2, and VDAC), which facilitates mitophagy. Ubiquitinated proteins, such as Mfn1 and Mfn2, then bind directly to the autophagy protein LC3, initiating mitophagy. Under stress conditions, mitophagy receptors like FUNDC1 and BNIP3 can directly cause mitochondrial fragmentation, promoting mitophagy. FUNDC1 recruits Drp1 to the mitochondrial outer membrane, where they interact to induce mitochondrial fission (49). Similarly, BNIP3 interacts directly with OPA1, leading to the disassembly of OPA1 oligomers and increased mitochondrial fragmentation in HeLa cells. At the onset of chemotherapy, mitophagy helps maintain normal cellular metabolism and inhibits tumor growth (50, 51). However, as chemotherapy progresses, enhanced mitophagy promotes tumor cell adaptation to the treatment. Abnormal activation of mitophagy supports the survival of cells with cancer stem cell-like properties, thereby contributing to chemotherapy resistance. In CRC cells, the BCL2 protein family members, such as BNIP3, NIX, and BCL2L13, play critical roles in regulating both apoptosis and mitophagy (52). An imbalance between the anti-apoptotic protein Bcl2 and the pro-apoptotic protein Bax is a key factor in enhancing chemotherapy resistance in tumor cells (53). After chemotherapy, CRC cells release large amounts of high-mobility group box 1 (HMGB1), which activates the HMGB1/RAGE/Erk signaling pathway. This induces the upregulation of LC3 and p62, promoting non-ubiquitin-dependent mitophagy and contributing to chemotherapy resistance in CRC cells (54). Additionally, elevated phosphorylation levels of Drp1Ser616 are associated with increased tumor recurrence risk and reduced survival time post-chemotherapy, highlighting the correlation between signaling pathways that promote mitophagy and chemotherapy resistance. In CRC patients treated with the chemotherapeutic agent cisplatin, Drp1 is highly expressed, and its role in inducing mitophagy further enhances chemotherapy resistance in CRC cells (55).

### 4.2 Mitochondrial-related metabolism promotes chemotherapy resistance in CRC

Cancer cells exhibit distinct metabolic heterogeneity compared to normal cells, adapting to high energy demands through enhanced glucose metabolism rather than oxidative phosphorylation (OXPHOS). This metabolic shift renders cancer cells less sensitive to chemotherapy agents targeting high-energy requirements. Research by Maddalena et al. identifies TRAP1, a molecular chaperone upregulated in CRC, as a promoter of chemotherapy resistance through the regulation of glycolytic metabolism (55). TRAP1 increases GLUT1 expression, glucose uptake, and lactate production while simultaneously reducing OXPHOS, thereby facilitating rapid adaptation of tumor cells to energy demands (56). TRAP1 interacts with phosphofructokinase-1 (PFK1) to maximize lactate production, supporting purine biosynthesis and enhancing DNA damage responses, mechanisms essential for chemotherapy resistance. Furthermore, the role of TRAP1 extends to promoting resistance to EGFR inhibitors; upregulation of TRAP1 or a high glycolytic metabolism state can diminish the efficacy of drugs like cetuximab (57). Thus, targeting TRAP1 or inhibiting the glycolytic pathway presents a potential strategy to overcome CRC chemotherapy resistance (58). Additionally, the serine metabolism pathway plays a crucial role in the chemotherapy resistance of CRC cells. In recent years, the problem of resistance to tumor chemotherapy has become increasingly prominent. Exploring the resistance mechanisms of chemotherapeutic drugs: [e.g., 5-fluorouracil (5-FU)] and how to improve the efficacy of chemotherapy is increasingly being addressed. It has been found that resistance of CRC cells to 5-FU is largely influenced by serine metabolism (57). CRC cells enhance chemoresistance by increasing endogenous serine synthesis or exogenous serine uptake while upregulating serine hydroxymethyltransferase-2 (SHMT2) expression. This promotes partitioning of one-carbon metabolism within mitochondria, increasing purine biosynthesis and DNA damage response, ultimately inducing chemoresistance in CRC cells (59, 60). Therefore, modulation of mitochondrial serine metabolism can effectively restore the sensitivity of CRC cells to 5-FU and improve the efficacy of treatment. The above study not only reveals the key mechanism by which serine metabolism promotes 5-FU resistance in CRC cells, but also provides important insights for the development of new therapeutic strategies targeting this metabolic pathway.

### 4.3 Mitochondrial dynamics modulate chemotherapy resistance in CRC

Mitochondrial fusion and fission are key dynamic processes that maintain the functional and structural integrity of mitochondria within the cell. Mitochondrial fusion is the process by which the outer and inner membranes of two or more mitochondria fuse to form a larger, more complete entity. Mitochondrial fusion promotes the repair of damaged mitochondria and maintains the integrity of mitochondrial DNA while balancing the internal environment of mitochondria (61). Mitochondrial fission is a process in which mitochondria split into two or more smaller entities due to the regulation of various factors. This helps to remove damaged or dysfunctional mitochondria, promote cell division, and adapt to changes in cellular energy requirements (62). The dynamic balance between mitochondrial fusion and division has been suggested to be a key factor in promoting chemotherapy resistance in CRC cells. CRC cells promote cell survival and growth by regulating the above processes to adapt to chemotherapy-induced stress. In CRC, tumor cells are helped to resist the toxic effects of chemotherapeutic drugs by increasing mitochondrial hyperfusion, which improves energy metabolism and resistance to cell death signals (63). On the other hand, in CRC, tumor cells will avoid apoptosis to the greatest extent possible by promoting mitochondrial fission, which allows tumor cells to more efficiently remove damaged mitochondria while The increased number of mitochondria due to fission can in turn meet the energy requirements for tumor cell growth and division (64).

Huang and colleagues found that the expression of ZNF746/PARIS, a substrate for the interaction between ZNF746 and Parkin, was significantly higher in CRC cells than in normal colorectal tissues, and that overexpression of ZNF746 inhibited the expression of proteins such as MFN1, MFN2, and PGC1α, which could disrupt the dynamic equilibrium between fusion and fission in the mitochondria and greatly reduce mitochondrial activity. This will disrupt the dynamic balance between mitochondrial fusion and fission, and ultimately lead to a significant reduction in mitochondrial activity. In addition, the imbalance of mitochondrial dynamics not only promotes the proliferation and progression of CRC cells, but also enhances their resistance to chemotherapeutic agents (e.g.5-FU) (65). It was further found that inhibition of ZNF746 protein expression significantly reduced the resistance of CRC cells to 5-FU, suggesting that the ZNF746 signaling pathway plays a key role in regulating CRC chemotherapeutic resistance. And when combined treatment with melatonin (Mel) and 5-FU was used, in line with the expected results. apoptosis of CRC cells was substantially increased, with an effect that far exceeded that of Mel or 5-FU alone. These findings emphasize the importance of mitochondrial fusion and fission in chemoresistance in CRC cells, and the fact that by modulating these processes they can be used as potential targets to overcome drug resistance in CRC cells (66). Inhibition of the ZNF746 signaling pathway, especially inhibition of the ZNF746 signaling target in combination therapy strategies, provides a key therapeutic strategy to reduce the resistance of CRC cells to chemotherapeutic agents. In addition, numerous studies have confirmed that miR-17-5p also plays a critical role in chemoresistance of CRC cells (67). In a mouse model of CRC, overexpression of miR-17-5p inhibits the efficacy of 5-FU and reduces the sensitivity of CRC cells to chemotherapeutic drugs. miR-17-5p-mediated chemotherapeutic resistance in CRC cells was found to be closely related to mitochondrial homeostasis by Sun and colleagues. In CRC, miR-17-5p directly binds to the 3’ untranslated region of Mitofusin 2 (MFN2), leading to abnormal mitochondrial dynamics through inhibition of mitochondrial fusion, enhancement of mitochondrial fission and autophagy, and thus promoting chemoresistance in tumor cells. Further studies revealed that methyltransferase-like protein 14 (METTL14) expression is significantly downregulated in CRC. Interestingly, the reduction of METTL14 expression promoted the expression of pri-miR-17 and miR-17-5p and led to a significant reduction of m6A levels in the cells. METTL14 regulates the m6A modification of cellular mRNA, inhibiting the recognition of specific sites by YTH domain containing 2 (YTHDC2) and disrupting the normal degradation of pri-miR-17. High levels of pri-miR-17, through the METTL14/miR-17-5p/MFN2 signaling pathway, greatly enhance the resistance of CRC cells to the chemotherapy drug 5-FU (3).

### 4.4 Enhanced mtDNA contributes to chemoresistance in CRC

The role of mitochondrial DNA (mtDNA) in chemotherapy resistance in CRC has garnered significant attention. MtDNA encodes proteins crucial for mitochondrial function, including several key proteins involved in the electron transport chain, directly impacting mitochondrial metabolic capacity and energy production (68). Therefore, any variation or damage to mtDNA may lead to mitochondrial dysfunction, thereby affecting cancer cells’ response to chemotherapy. The main mechanisms linking mtDNA and chemoresistance are as follows:1. MtDNA mutations and resistance: Specific mtDNA mutations may promote cancer cell tolerance to chemotherapy drugs by altering mitochondrial metabolic pathways, increasing energy production efficiency, allowing cancer cells to survive more effectively under drug exposure (69).2. Mitochondrial bioenergetic alterations: MtDNA damage or mutations can lead to changes in mitochondrial bioenergetics, such as increased oxidative phosphorylation (OXPHOS) or alterations in glycolytic pathways, which may enhance cancer cell survival and confer resistance to chemotherapy (70).3. Mitochondria-mediated apoptosis suppression: Mitochondria play a crucial role in regulating apoptosis (71). Mutations in mtDNA may interfere with apoptotic pathways, such as by reducing the release of apoptotic proteins, thereby diminishing chemotherapy-induced cell death. Research by Mou et al. found that a decrease in mtDNA levels inhibits apoptosis and enhances aerobic glycolysis, leading to chemotherapy resistance in CRC (72).

**Figure 1:**
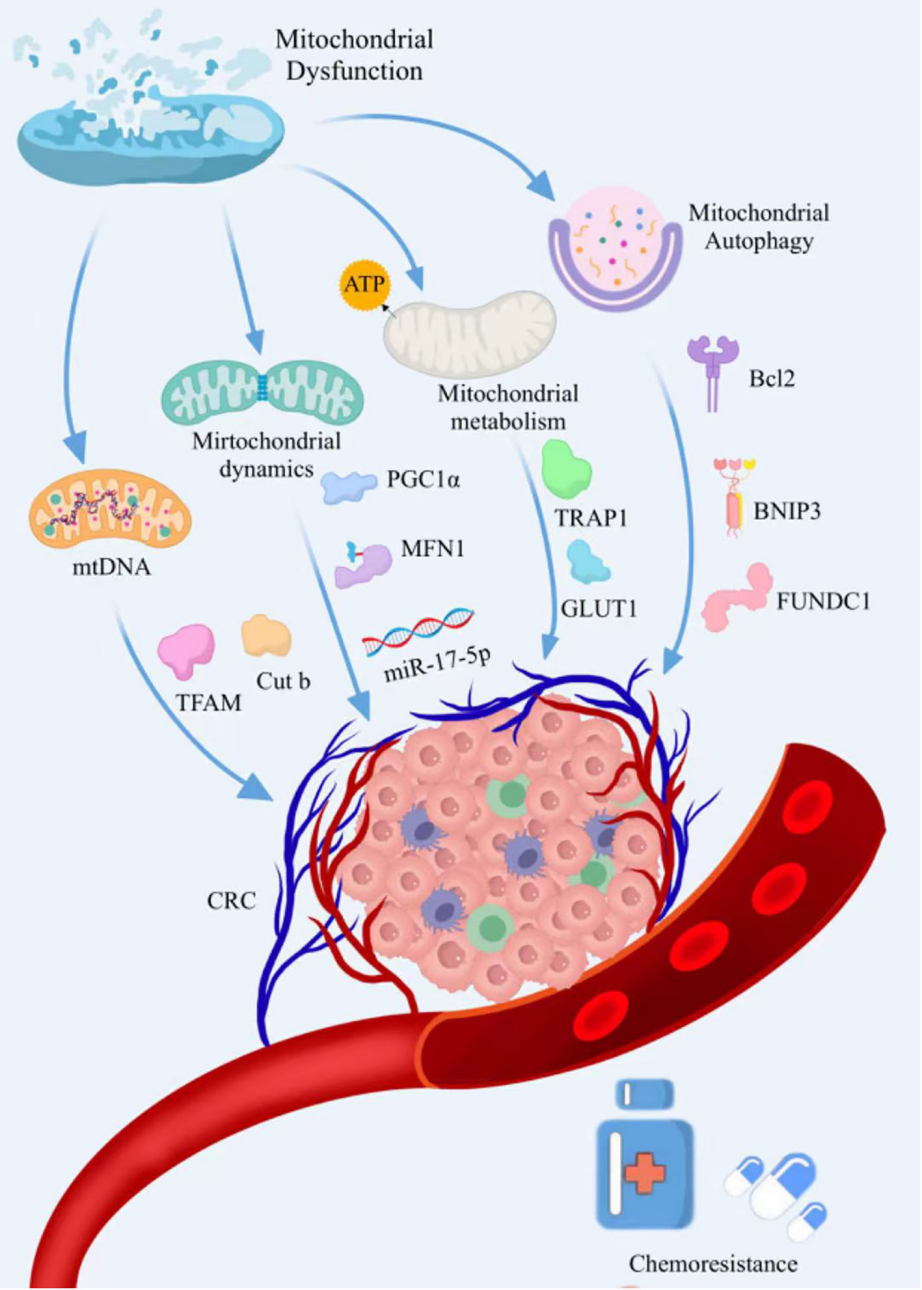
The mechanisms by which mitochondrial dysfunction drives the development of chemoresistance in CRC. Mitochondrial dysfunction induces drug resistance in CRC cells by regulating mitochondrial dynamics, mitochondrial autophagy, mitochondrial metabolism, and mtDNA.

Additionally, mitochondrial transcription factor A (TFAM) plays a key role in the replication and transcription of mtDNA. Currently, the specific mechanisms of TFAM in the development and progression of CRC remain largely unknown. Studies have confirmed that in CRC with microsatellite instability (MSI), frameshift mutations frequently occur in the nucleotide sequences encoding TFAM, but similar mutations are not found in microsatellite stable (MSS) CRC (73). Further research has shown that in MSI CRC, the high expression of truncated mutation of TFAM significantly reduces the expression of TFAM protein in cells and is positively correlated with mtDNA depletion. Additional studies in mouse models of CRC with RKO cells having TFAM truncation mutations showed that forcibly upregulating the expression of TFAM protein in these cells leads to a significant secretion of cytochrome b (Cyt b) and a higher sensitivity to apoptosis induced by chemotherapy drugs (such as cisplatin). Digging deeper into the specific mechanism revealed that RKO cells with truncated mutation TFAM showed reduced binding to HSPs, leading to decreased transcription of Cyt b and destabilization of mitochondria, which triggers significant proliferation of tumor cells and strong resistance to chemotherapy drugs. However, when TFAM protein expression is increased, the transcription of Cyt b is restored to normal, and the proliferation of tumor cells is significantly inhibited (74). In MSI CRC, the high incidence of abnormal mutations of TFAM directly leads to a reduction in mtDNA copy number, thereby destroying mitochondrial stability. These mutations almost exclusively occur in MSI CRC and greatly promote the resistance of tumor cells to chemotherapy drugs (such as cisplatin) (75). Here, we have summarized the mechanisms by which mitochondrial dysfunction drives the development of chemoresistance in CRC (**Figure 1**).

## 5 Targeting mitochondria to overcome chemoresistance

In the realm of CRC therapy, chemoresistance significantly challenges the efficacy of chemotherapy protocols, culminating in treatment failures. In recent years, there has been an increasing focus on therapeutic strategies targeting mitochondria to circumvent tumors’ resistance to conventional chemotherapy agents (77). Playing a pivotal role in cell death, mitochondria influence the fate of tumor cells by regulating energy metabolism, apoptosis, and survival signals (34, 78). Thus, strategies targeting mitochondria offer a promising avenue to augment the effectiveness of chemotherapy drugs, particularly in tumor cells that have developed drug resistance.

### 5.1 Targeting the mitochondrial ETC to alleviate chemoresistance

Targeting mitochondrial mechanisms to overcome chemoresistance has emerged as a cutting-edge strategy in CRC treatment. Complex I (CI), as one of the most crucial components of the mitochondrial electron transport chain (ETC), transfers electrons from NADH, produced in the tricarboxylic acid cycle (TCA), to ubiquinone (UbQ), thereby maintaining the proton gradient across the mitochondrial inner membrane (MIM) (76, 77). Research has demonstrated that inhibitors directly targeting CI, such as metformin and piericidin, could serve as potential anti-tumor treatments. The research by Tang et al. found that the SLIT-ROBO Rho GTPase-activating protein 2 (srGAP2) can bind to and directly interact with mitochondrial Complex I (CI), enhancing its activity, which in turn inhibits the sensitivity of CRC cells to chemotherapy. Therefore, targeting the suppression of SRGAP2 expression in CRC cells, by inhibiting the function and activity of mitochondrial CI, could weaken mitochondrial respiration and increase the sensitivity of CRC cells to chemotherapy. Inhibition of SRGAP2 expression in mitochondria directly leads to a decrease in CI activity, which inhibits the resistance of CRC cells to chemotherapeutic drugs. Targeting SRGAP2 with the aim of increasing the sensitivity of CRC cells to chemotherapy may be one of the key strategies to be used as a future strategy to ameliorate chemotherapy resistance in CRC cells (78) In addition, it was found that: metformin could alleviate the problem of chemoresistance in CRC cells by inhibiting CI, while substantially enhancing the ability to kill tumors (79). Metformin has proven to enhance the effects of chemotherapeutic agents such as cisplatin, doxorubicin (Dox), and 5-FU in numerous preclinical and clinical studies. Recent clinical trials observed that the combination of metformin with 5-FU significantly improves the progression-free survival and overall survival of patients with refractory CRC (80). These findings provide a scientific basis for integrating mitochondrial-targeted therapies to overcome resistance in human lymphomas, showcasing the targeting of mitochondria as a potent strategy to surmount cancer treatment resistance.

### 5.2 Targeting mitophagy to improve chemoresistance

**Figure 2:**
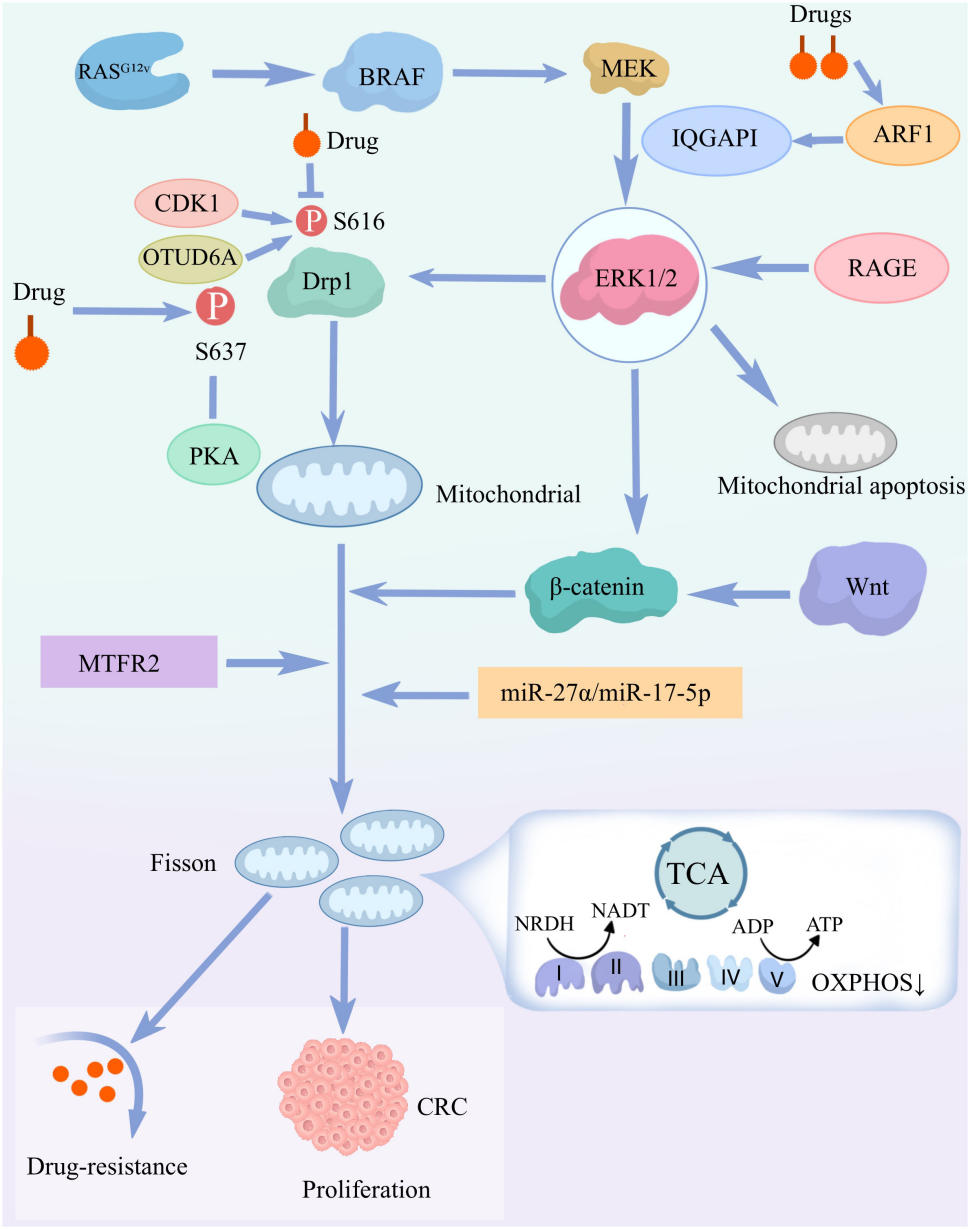
Mechanisms by which mitochondrial dysfunction drives the development of chemotolerance in colorectal cancer (CRC): mitochondrial dysfunction drives CRC cell proliferation and chemotolerance. This is mainly through the activation of the signaling pathways and molecular mechanisms described above. RASG 1 mutations activate the BRAF-MEK-ERK1/2 signaling pathway, leading to phosphorylation of Drp1 (S616), which induces mitochondrial fission and cell proliferation. ERK 1/2 signaling regulates mitochondrial apoptosis through RAGE and IQGAP1.Mitochondrial fission and dysfunction through pathways involving β-catenin, miR-27α/miR-17-5p, and MTFR2 would further promote CRC cell proliferation and chemotolerance. Drugs also affect Drp1 activity through multiple pathways, including CDK1, OTUD6A and PKA-mediated phosphorylation. Further enhancement of the above signaling pathway expression.

Mitophagy is a cellular process that removes damaged mitochondria, contributing to the stability of mitochondrial quality within cells. By activating mitophagy, dysfunctional mitochondria can be cleared, preventing their support for tumor cell survival through metabolic reprogramming, thereby restoring or enhancing tumor cell sensitivity to chemotherapeutic drugs (81, 82). Research has discovered that in CRC cells, highly phosphorylated nitric oxide synthase 3 (NOS3) is crucial for tumor cells to resist oxaliplatin. Further studies found that when oxaliplatin is used in combination with cannabidiol (CBD), it significantly reduces the phosphorylation levels of NOS3 in tumor cells and leads to the production of excess reactive oxygen species (ROS), thereby inducing autophagy in CRC cells and enhancing their sensitivity to oxaliplatin (83). Based on a randomized clinical trial (NCT03607643), this trial is evaluating the effects of combining CBD with oxaliplatin in treating CRC. This suggests that by reducing the phosphorylation level of NOS3 and inducing mitochondrial dysfunction to produce excessive reactive ROS, CBD may help overcome resistance to oxaliplatin, thereby activating the autophagy process (84) (**Figure 2**).

### 5.3 Targeting mitochondrial dynamics to overcome chemoresistance

Mitochondrial dynamics, encompassing mitochondrial fission and fusion, are crucial processes that play a key role in maintaining the integrity of mitochondrial function and structure (85, 86). During the processes of mitochondrial fission and fusion, proteins such as DRP1, MFN1, and MFN2 act to enhance the effectiveness of chemotherapy by inhibiting the metabolic pathways and proliferation of tumor cells (87). In mouse models of CRC, the interaction between zinc finger protein 746 and Parkin disrupts the dynamic balance between mitochondrial fission and fusion, and by inhibiting the expression of proteins such as MFN1, MFN2, and PGC1α, significantly suppresses mitochondrial activity, thereby promoting resistance of CRC cells to chemotherapy. The combined use of melatonin (Mel) and 5-FU, by inhibiting ZNF746-mediated signaling, significantly improves the chemoresistance of CRC to 5-FU (88). Here, we have summarized the mechanisms related to targeting mitochondria for overcoming the development of chemoresistance in CRC (**Table 1**).

**Table 1:** The mechanisms related to targeting mitochondria for overcoming the development of chemoresistance in CRC.

Section	Target	Mechanism	Outcome	Ref
Targeting the mitochondrial ETC to alleviate chemoresistance	Complex I (CI) of ETC	Inhibitors like metformin, affecting CI activity and SRGAP2 interaction	Reduced resistance, improved chemotherapeutic efficacy	(60, 62)
Targeting Mitophagy to Overcome Chemoresistance	Damaged mitochondria via mitophagy	Clearing dysfunctional mitochondria, reducing NOS3 phosphorylation with CBD	Overcoming oxaliplatin resistance	(63, 66)
Targeting mitochondrial dynamics to improve chemoresistance	Mitochondrial fission and fusion proteins (DRP1, MFN1/2)	Disrupting ZNF746/PARIS-mediated inhibition of dynamics	Improved chemoresistance to 5-FU	(67, 69)

### 5.4 Multi-omics reveal mechanisms of action associated with CRC occurrence and development

Multi-omics analysis techniques, including genomics, transcriptomics, proteomics, and metabolomics, have revealed the multifaceted roles of mitochondria in cancer. Genomic analysis has unveiled mutations and rearrangements in mitochondrial DNA, which may facilitate tumor onset and progression (89). Transcriptomic studies, particularly through RNA sequencing (RNA-seq), have demonstrated the link between mitochondrial dysfunction and the metabolic reprogramming of cancer cells (90). These multimodal approaches offer a comprehensive perspective on how mitochondria promote tumor development by affecting the metabolism, proliferation, and immune evasion of cancer cells. Genomics and transcriptomics, especially single-cell sequencing techniques, enable the tracking of cellular lineages in humans by detecting mitochondrial mutations. This approach reveals variations in mtDNA at the single-cell level, offering new tools for studying cell origins, tumorigenesis, and mutations. Ludwig et al., through single-cell RNA or ATAC sequencing, could detect mtDNA mutations in CRC, allowing for lineage inference and the prediction of mitochondrial transfer between cells (91). Moreover, systematic studies on mitochondrial transfer between cancer cells and T cells have been detailed using single-cell sequencing. Research has found that under specific conditions, cancer cells can transfer their mitochondrial DNA to T cells, potentially affecting T cell function, including their activity and survival in the tumor microenvironment (92). This finding offers a new perspective on tumor immune evasion mechanisms and may provide a theoretical basis for developing new cancer treatment strategies. Metabolomic studies have revealed alterations in mitochondrial metabolic pathways in cancer cells, supplying energy and biosynthetic precursors. Metabolomics has revealed the key role of mitochondrial metabolism in tumor development, especially through metabolic reprogramming to support the proliferation, survival, and progression of tumor cells (93). For instance, mutations in mitochondrial isocitrate dehydrogenase (IDH) have been found in various human cancers, including CRC, affecting tumor cell metabolism and epigenetic regulation by producing 2-hydroxyglutarate (2HG) (94). Additionally, it has been found that tumor cells often undergo mitochondrial genomic variations that change cellular bioenergetics rather than hindering metabolism, which can further drive cancer by altering retrograde signaling, regulating cellular signals, epigenetic modifications, chromosomal structures, and transcription mechanisms. Proteomic analysis provides direct evidence of changes in mitochondrial protein expression that affect the growth, survival, and death of tumor cells. Proteomics, by providing a comprehensive characterization of mitochondrial protein functions, enhances our understanding of the molecular interactions of mitochondrial proteins in cancer development (95). This approach will uncover more biomarkers for diagnosis and prognosis, and improve the treatment outcomes for cancer patients. Xia and colleagues, in their study on CRC, discovered through quantitative proteomics analysis that β-citrate (EA) mainly targets mitochondrial ribosomal proteins, which are usually upregulated in CRC patients. EA inhibits the cell cycle at low concentrations by regulating CDK1, CDK6, and CDC20, and at high concentrations, it affects iron metabolism-related proteins, such as lowering ferritin (FTL) and raising transferrin (TF), ceruloplasmin (CP), and acyl-CoA synthetase long-chain family member 4 (ACSL4), inducing ferroptosis in cells (96).

### 5.5 Challenges and future prospects

Current Challenges: The first thing that needs to be considered when trying to utilize targeted mitochondrial therapy for CRC in the future is the heterogeneity of CRC. CRC varies significantly between patients and may also present different biological characteristics in different tumor regions of the same patient. This heterogeneity may affect mitochondrial function, metabolic status, and response to therapy. Therefore, a single mitochondria-targeted therapeutic regimen may not be applicable to all patients, and more individualized treatment strategies are needed. Targeting mitochondrial therapy requires an effective drug delivery system. Since mitochondria are located in the cytoplasm and have a dual-membrane structure, drugs need to cross both the cellular and mitochondrial membranes to be effective. However, existing delivery systems (e.g., liposomes, nanoparticles, etc.) ensure precise delivery of drugs to mitochondria, but may pose problems such as toxic side effects or unstable drug release. Integration and Analysis of Mitochondrial Data: Data integration is a complex process that is extremely difficult due to the different data sources, sequencing methods, manufacturers, and batches involved. Analyzing multimodal data is also a major challenge. Currently developed computational tools and algorithms are far from sufficient to extract complete and valuable biological information, and it is difficult to form a unified and complete explanation of complex biological processes at a later stage. Biomarker identification and validation: The identification and validation of potential mitochondria-related biomarkers requires large sample sets and extensive studies in multiple independent cohorts to ensure their validity and accuracy for clinical use. Interdisciplinary Collaboration and Communication: In-depth exploration of the role of mitochondrial genomics in CRC will require interdisciplinary collaboration including: computer science, bioinformatics, molecular biology, and clinical medicine. Interdisciplinarity is a key direction for the future. By exploring mitochondrial multi-omics analysis, we are gradually revealing how mitochondria regulate tumor cell progression in CRC by regulating cellular energy metabolism and death pathways. We have reason to believe that the future development and depth of multi-omics technology will provide a solid theoretical foundation for the treatment of mitochondria in CRC. Multimodal analysis has offered a comprehensive perspective on the role of mitochondria in CRC, employing an integrative approach that includes genomics, transcriptomics, proteomics, and metabolomics. This methodology has uncovered how mitochondria influence CRC cell processes such as energy metabolism, apoptotic pathways, and drug resistance, enriching our understanding of mitochondrial roles in tumor progression and unveiling potential avenues for novel therapeutic strategies.

Future Perspectives: Refined Mechanistic Studies: Further delineation of precise molecular mechanisms of mitochondria in CRC development is required, focusing on mitochondrial dynamics changes, mitochondrial DNA mutations, and the connection between mitochondria and cell death mechanisms. Therapeutic Target Development: New therapeutic drugs or interventions targeting mitochondrial-related molecular pathways, as revealed by multimodal data, are to be developed to enhance CRC treatment outcomes. Combination Therapy Strategies: Investigating the integration of mitochondrial function modulation with conventional treatments, such as chemotherapy and radiotherapy, aims to overcome the limitations of single treatment modalities and enhance efficacy. Individualized treatment regimens: Designing individualized treatment regimens based on different pathological and biological characteristics of CRC patients (e.g., unique mitochondrial status and function) to achieve more precise treatment.

## 6 Conclusion

Mitochondrial dysfunction leads to cellular metabolic disorders, abnormal apoptosis, and increased oxidative stress, which in turn promotes tumorigenesis and progression. Mitochondria are also central to the formation of drug resistance mechanisms in tumor cells, and all of the above pathways affect the response and tolerance of CRC cells to chemotherapy to some extent. Therefore, it is crucial to deeply explore the mechanisms of how mitochondrial dysfunction in CRC cells drives tumor cell genesis and proliferation. As mitochondrial multi-omics studies have progressed, it has become increasingly straightforward and feasible to probe the specific mechanisms underlying mitochondrial biogenesis, mitochondrial DNA mutations, and mitochondrial fission and fusion processes. Currently, clinical trials are exploring mitochondrial function as a therapeutic target for overcoming chemoresistance in CRC cells, and preliminary results have been achieved. Meanwhile, multi-omics technologies are gradually revealing the key role of mitochondria. In the future, therapeutic strategies targeting mitochondria will be the key to improving chemoresistance in CRC cells, as well as to significantly improving the quality of survival of CRC patients.

## Glossary

CRC: Colorectal Cancer
ETC: Electron Transport Chain
CI: Complex I
UbQ: Ubiquinone
MIM: Mitochondrial Inner Membrane
SRGAP2: SLIT-ROBO Rho GTPase-activating Protein 2
NOS3: Nitric Oxide Synthase 3
CBD: Cannabidiol
ROS: Reactive Oxygen Species
RNA-seq: RNA Sequencing
mtDNA: Mitochondrial DNA
TCA: Tricarboxylic Acid Cycle
5-FU: 5-Fluorouracil
PFK1: Phosphofructokinase-1
SHMT2: Serine Hydroxymethyltransferase 2
OXPHOS: Oxidative Phosphorylation
HMGB1: High Mobility Group Box 1
RAGE: Receptor for Advanced Glycation End-products
Drp1: Dynamin-related Protein 1
PINK1: PTEN-induced Putative Kinase 1
LC3: Microtubule-associated Protein 1A/1B-light Chain 3
BAX: Bcl-2-associated X Protein
BCL2: B-cell Lymphoma 2
VDAC: Voltage-dependent Anion Channel
FUNDC1: FUN14 Domain Containing 1
BNIP3: BCL2/adenovirus E1B 19kDa-interacting Protein 3
OPA1: Optic Atrophy 1
TFAM: Mitochondrial Transcription Factor A
Cyt b: Cytochrome b
EA: β-citrate
FTL: Ferritin Light Chain
TF: Transferrin
CP: Ceruloplasmin
ACSL4: Acyl-CoA Synthetase Long-Chain Family Member 4
m6A: N6-methyladenosine
YTHDC2: YTH N6-methyladenosine RNA Binding Protein 2
METTL14: Methyltransferase-like Protein 14
miR-17-5p: MicroRNA-17-5p
ZNF746: Zinc Finger Protein 746
PGC1α: Peroxisome Proliferator-activated Receptor γ
MSI: Microsatellite Instability
MSS: Microsatellite Stable
HSPs: Heat Shock Proteins.
